# Quality, Usability, and Effectiveness of mHealth Apps and the Role of Artificial Intelligence: Current Scenario and Challenges

**DOI:** 10.2196/44030

**Published:** 2023-05-04

**Authors:** Alejandro Deniz-Garcia, Himar Fabelo, Antonio J Rodriguez-Almeida, Garlene Zamora-Zamorano, Maria Castro-Fernandez, Maria del Pino Alberiche Ruano, Terje Solvoll, Conceição Granja, Thomas Roger Schopf, Gustavo M Callico, Cristina Soguero-Ruiz, Ana M Wägner, WARIFA Consortium

**Affiliations:** 1 Endocrinology and Nutrition Department Complejo Hospitalario Universitario Insular Materno Infantil Las Palmas de Gran Canaria Spain; 2 Complejo Hospitalario Universitario Insular - Materno Infantil Fundación Canaria Instituto de Investigación Sanitaria de Canarias Las Palmas de Gran Canaria Spain; 3 Research Institute for Applied Microelectronics Universidad de Las Palmas de Gran Canaria Las Palmas de Gran Canaria Spain; 4 Instituto Universitario de Investigaciones Biomédicas y Sanitarias Universidad de Las Palmas de Gran Canaria Las Palmas de Gran Canaria Spain; 5 Norwegian Centre for E-health Research University Hospital of North-Norway Tromsø Norway; 6 Faculty of Nursing and Health Sciences Nord University Bodø Norway; 7 Departamento de Teoría de la Señal y Comunicaciones y Sistemas Telemáticos y Computación Universidad Rey Juan Carlos Madrid Spain; 8 See Acknowledgements Tromsø Norway

**Keywords:** artificial intelligence, chronic disease prevention and management, big data, mobile health, mHealth, noncommunicable diseases, mobile phone

## Abstract

The use of artificial intelligence (AI) and big data in medicine has increased in recent years. Indeed, the use of AI in mobile health (mHealth) apps could considerably assist both individuals and health care professionals in the prevention and management of chronic diseases, in a person-centered manner. Nonetheless, there are several challenges that must be overcome to provide high-quality, usable, and effective mHealth apps. Here, we review the rationale and guidelines for the implementation of mHealth apps and the challenges regarding quality, usability, and user engagement and behavior change, with a special focus on the prevention and management of noncommunicable diseases. We suggest that a cocreation-based framework is the best method to address these challenges. Finally, we describe the current and future roles of AI in improving personalized medicine and provide recommendations for developing AI-based mHealth apps. We conclude that the implementation of AI and mHealth apps for routine clinical practice and remote health care will not be feasible until we overcome the main challenges regarding data privacy and security, quality assessment, and the reproducibility and uncertainty of AI results. Moreover, there is a lack of both standardized methods to measure the clinical outcomes of mHealth apps and techniques to encourage user engagement and behavior changes in the long term. We expect that in the near future, these obstacles will be overcome and that the ongoing European project, Watching the risk factors (WARIFA), will provide considerable advances in the implementation of AI-based mHealth apps for disease prevention and health promotion.

## Introduction

Chronic, noncommunicable diseases (NCDs) are the main cause of death and morbidity worldwide and have important social and economic effects. The 4 leading NCDs are cardiovascular disease, chronic respiratory disease, cancer, and diabetes, all of which share 4 behavioral risk factors: tobacco, alcohol, unhealthy diet, and physical inactivity [1].

The Global Burden of Disease 2019 program assessed 369 health conditions in 204 countries and regions. The incidence, prevalence, mortality, years of life lost, years of life with disability, and disability-adjusted life years were recorded and compared over time. From 1990 onwards, there has been a shift toward a higher proportion of burden caused by years of life with NCD-related disabilities. The top 10 diseases affecting disability-adjusted life years for adults aged 50-74 years in 2019 included 9 NCDs: ischemic heart disease, stroke, chronic obstructive pulmonary disease, diabetes, lung cancer, chronic kidney disease, age-related hearing loss, low back pain, and cirrhosis [2].

The Global Burden of Disease 2019 program also estimated the attributable mortality, years of life lost, years of life with disability, and disability-adjusted life years for 87 risk factors and combinations of risk factors. The risk factors that accounted for the highest number of deaths were high systolic blood pressure (19% of all deaths) and tobacco use (15.4% of deaths) [3]. The top modifiable risk factors in adults also included high fasting plasma glucose, high BMI, high low density lipoprotein cholesterol, alcohol use, ambient particulate matter, low whole grain intake, and high sodium intake. These data reveal the importance of lifestyle risk factors for NCDs.

**Figure 1:**
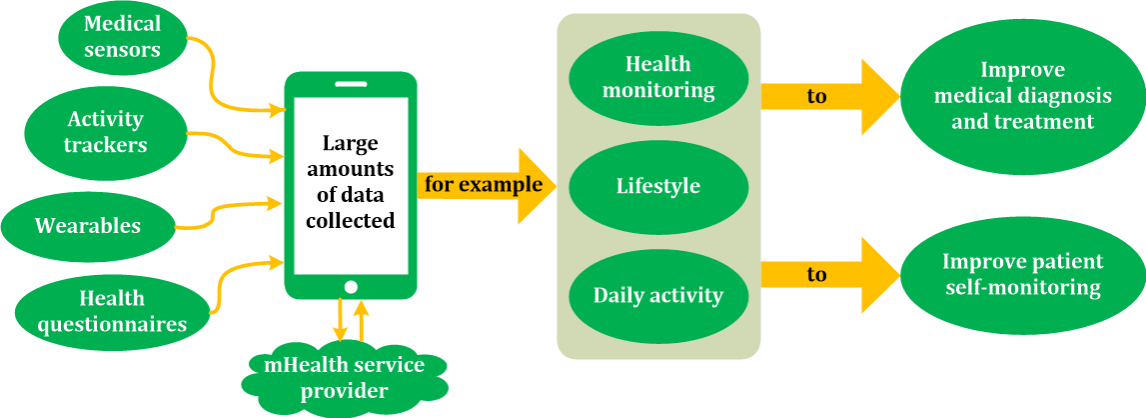
Conceptual block diagram of mobile health (mHealth) app function.

Mobile health (mHealth) apps are widely accessible, low in cost, and can help promote healthy behaviors. Figure 1 shows how mHealth apps work, where data from different sources are combined in a smartphone-based app to provide guidance and advice for health care professionals, healthy individuals, and patients. According to a systematic review of 52 randomized controlled trials, a notable body of evidence indicates that mHealth apps can promote healthy food choices (increasing the consumption of vegetables and reducing salt intake), increase in physical activity (assessed as the number of daily steps), and reduction of sedentary time [4]. For people with diabetes, especially type 2 diabetes, the use of an mHealth app has been associated with a moderate improvement in glycemic control (reduction of 0.2%-0.4%—2-4 mmol/mol—in HbA1c) [5]. However, many widely used mHealth apps are not evidence-based, are more disease-centered than person-centered, have limited usability, and are associated with serious concerns regarding user privacy [6].

The use of artificial intelligence (AI) to develop mHealth apps is being explored with the aim of providing more personalized health care [7,8], preventing diseases, improving treatment, remotely monitoring individuals with chronic diseases, providing better health care delivery, and decreasing costs and time to diagnosis [9]. However, several obstacles limit the use of AI-based mHealth apps in medical practice [10,11]. Here, we briefly summarize the current scenario and challenges facing mHealth apps and the implementation of AI algorithms, with a special focus on NCD prevention and management. In addition, we recommended possible solutions to these problems according to our experience in the ongoing European project “Watching the risk factors (WARIFA): Artificial intelligence and the personalized prevention and management of chronic conditions*.”* WARIFA aims to address the challenges facing mHealth through the development of an AI-based app to empower individuals to prevent and manage NCDs.

Although an individual analysis of the frameworks used to manage each NCD in each participating territory in the WARIFA project could be useful for understanding the problems and possible solutions arising in the project, such considerations are beyond the scope of this paper. However, the following three aspects should be highlighted: (1) the 4 NCDs that the project intends to tackle are the main causes of death and morbidity worldwide and share 4 behavioral risk factors: *tobacco consumption*, *alcohol consumption*, *unhealthy* *diet*, and *physical inactivity*; (2) in particular, 2 specific diseases are the main targets of the project: *type 1 diabetes* and *skin cancer*. This decision is guided by the fact that they are also clear examples of conditions highly influenced by behavioral factors; (3) The broad spectrum of conditions tackled, the heterogeneity of potential end users, and the peculiarities of each country participating in the WARIFA project are constantly creating challenges that the developing team needs to deal with. Some of these will be explored later in this paper.

### Quality of mHealth Apps

#### Rationale and Guidelines for Implementation

Interest in mHealth apps is steadily increasing. However, despite a growing body of evidence, their effectiveness is still a subject of controversy. In general, apps have been found to exert a small but notable effect on enhancing healthy lifestyles, specifically regarding nutrition [12], physical activity [13], management of chronic conditions [14-17] and mental health [18]. However, the underlying mechanisms by which some, but not all, apps induce these behavior changes are not fully understood [19].

In 2019, the World Health Organization published extensive guidelines on implementing digital interventions, including mHealth apps, based on a review of the existing evidence [20]. Their recommendations affect infrastructure, the health workforce, governance, financial resources, interoperability, and standards, in addition to policy and regulations (Textbox 1).

The design and implementation of mHealth apps should also be guided by the 9 Principles for Digital Development [21]: design with the user; understand the existing ecosystem; design for scale; build for sustainability; be data-driven; use open standards, open data, open source, and open innovation; reuse and improve; address privacy and security; and be collaborative. Although these principles are not legal requirements, they provide a framework for the design and development of mHealth apps.

**World Health Organization recommendations for the implementation of digital interventions, including mobile health apps.**

**Recommendations**

- Involve stakeholders in program design and implementation
- Assess efficient integration of programs within the health system
- Secure data confidentiality and to obtain informed consent
- Ensure health workers have adequate training, supervision, support, and incentives
- Ensure access to network connectivity and electricity
- Ensure health workers have access to functioning digital devices

#### Legal Requirements

To guarantee safety, effectiveness, and privacy, mHealth apps and other AI-based health solutions must meet certain legal requirements.

##### US Requirements

Four main federal laws regulate mHealth apps in the United States. The Health Insurance Portability and Accountability Act protects the *privacy and security* of health information and requires certain entities to provide notifications of health information breaches. It is enforced by the Office for Civil Rights, within the US Department of Health & Human Services. The Federal Food, Drug, and Cosmetic Act regulates the *safety and effectiveness of medical devices*, including certain mHealth apps. The Food and Drug Administration focuses its regulatory oversight on a small subset of mHealth apps that pose a higher risk if they do not work as intended. The Federal Trade Commission (FTC) Act *prohibits deceptive or unfair practices*, including those involving false or misleading claims regarding app safety or performance. The FTC’s Health Breach Notification Rule requires certain businesses to *provide notifications following breaches of personal health record information*. Depending on the specific characteristics of each mHealth app, one or more regulatory bodies may be involved. A web-based tool is available to guide this process [22].

##### European Union Requirements

In the European Union (EU) and European Economic Area, mHealth apps and AI-based health solutions are considered medical devices and need to meet the corresponding requirements [23]. Medical devices are classified according to their level of risk into class I, IIa**,** IIb, and III (low, medium, medium-high, and high risk, respectively). General safety requirements include, at minimum, clinical or performance evaluations. The manufacturer of the device must present a clinical evaluation report that includes a review of relevant scientific literature, results of available investigations, and alternative treatment options. However, there has been limited clinical investigation of AI-based medical devices, and alternative treatment options often involve models and data that are trade secrets and are not available for comparison. The clinical evaluation must include 3 components: first, at least one analyte associated with a clinical condition; second, the ability of the medical device to detect and measure the analyte accurately; and third, the ability of the device to yield valid and usable results relating to a specific clinical condition. Finally, four factors determine whether there is sufficient clinical evidence to warrant the approval of the device: *intended use*, *side effects*, *interferences* or *cross-reactions of the device*, and the *risk:benefit ratio*.

The most recent EU regulation on medical devices (Regulation EU 2017/745) was enacted on May 26, 2021. Several guidelines published by the European Commission complement this regulation [24,25]. Apps included within the framework of medical devices must meet the objective of influencing people’s health, avoid handling population data, and comply with medical device regulations. The regulation also includes the establishment of a medical device database to improve transparency for both patients and health care providers.

Before the recent EU regulation took effect, there had been many attempts to standardize the assessment of mHealth app quality. Indeed, both public and patient-led organizations have developed certification programs, as reviewed in the European (H2020) Innovation and Knowledge mHealth Hub [26,27]. If the app meets a set of pre-established requirements, it is given a quality seal or certification, which in turn increases user confidence in the app. However, most of these certification programs are developed and maintained at the national or regional level, often in the national language and on a voluntary basis, which limits their adoption and integration in other health care systems.

#### Assessing Quality of mHealth Apps

Several groups have proposed instruments to assess mHealth apps, each of which focuses on slightly different aspects of quality, such as privacy, technical stability, user experience, usability, accessibility, data (collection, sharing, user rights, and security), professional assurance, and main app functions (Textbox 2). Several of these initiatives were included in a recent comprehensive review of health apps [28].

Another key aspect of quality assurance is *validation*. Recently, a “V3 validation framework” for Biometric Monitoring Technologies (BioMeTs) was proposed by Goldsack et al [29]. The framework includes an analysis of 3 key aspects of validation: *verification*, *analytical validation,* and *clinical validation*. *Verification* is concerned with evaluating the performance of sensor technology within a BioMeT and the sample-level data it generates against a prespecified set of criteria. *Analytic validation* deals with the performance of the algorithm and the ability of BioMeT to measure, detect, or predict physiological or behavioral metrics. *Clinical validation* addresses the question of whether a BioMeT acceptably identifies, measures, or predicts a meaningful clinical, biological, physical or functional state, or experience in the stated context of use, including within a specific population. Although BioMeTs and mHealth apps are different concepts, they overlap to a great extent. We recommend this V3 validation framework as a useful model for guiding the validation of health apps.

**Specific aspects of mobile health (mHealth) quality evaluated with different assessment tools.**

**Mobile App Rating Scale (MARS)**

- Engagement, functionality, aesthetics, information, subjective quality, and perceived impact on knowledge, attitudes and behavior of the user.

**App Behavior Change Scale (ABACUS)**

- A total of 21 yes or no questions addressing 4 main topics: knowledge and information, goals and planning, feedback and monitoring, and actions taken.

**Health on the Net (HoN) Foundation [30] code**

- Eight principles related to quality certification of websites: authorship, complementarity, confidentiality, attribution, and guarantee and transparency of authors, funds, and advertising policy. There is a controversy regarding whether the foundation has the means to certify that the principles continue to be followed after their initial evaluation.

**mHealth service quality scale [31,32]**

- User-perceived quality of the platform, interaction, and outcome in terms of system reliability, efficiency, availability, flexibility, privacy, responsiveness, assurance, empathy, functional and emotional benefits, and control variables (age, sex, income, cost, experience, and trust). Service quality affects user satisfaction, intention to continue using the system, and quality of life.

**Organization for the Review of Care and Health Apps (ORCHA) [33]**

- Data management (data collection, sharing and security, user rights, and compliance with data management standards), professional assurance (professional backing, evidence, and regulatory requirements), usability and accessibility (app design standards, user engagement, accessibility, usability, customer support, and user feedback), and the main functions of the app (specific mechanics and features built into the app, such as signposting services, remote clinical monitoring, goal setting, and gamification). Specific and average scores (0-100) are calculated.

**Healthy Living Apps [34]**

- Functionality (using MARS) and behavior change effectiveness (using ABACUS) with a 5-star grading system. An overall score is obtained by averaging these two.

**DISCERN instrument [35,36]**

- Originally designed to evaluate physical documents for patients but can be adapted to evaluate any information delivered by text. Three sections addressing reliability (8 questions), content (7 questions), and an overall quality rating (one question).

### Usability of mHealth Apps

#### Definitions

The International Organization for Standardization (ISO) defines usability as the extent to which a system, product, or service can be used by specified users to achieve specified goals with *effectiveness* (accuracy and completeness with which users achieve their goals), *efficiency* (resources used in relation to the results achieved), and *satisfaction* (measure of the extent to which the user’s needs, expectations, and preferences [NEPs]) are met because of interaction with the system) in a specified context of use [37]. Another accepted definition of usability was proposed by Nielsen [38] and includes 5 dimensions: *learnability*, that is, the ease of learning the functionality and behavior of the system; *efficiency*, defined as the level of productivity of the user after learning the system; *memorability*, the ease of remembering the functionality of the system; *error management*, which measures the capability of the system to help users make fewer mistakes and correct any mistakes they make; and *satisfaction*, a measure of how pleasant the system is to use.

#### Features Associated With Increased Usability

Several features have been associated with higher usability of mHealth apps [39-46]: a simple and intuitive interface (using clear graphs and instructions or widely known symbols instead of text), understandable and actionable tasks, minimum manual input (automatically recording location with a GPS tracker or heart rate with a smartwatch), assistance if needed to start using the app, and in-app educational content. A literature review (Textbox 3) was performed to assess randomized controlled trials, systematic reviews, and meta-analyses dealing with the assessment of mHealth in terms of usability, engagement, and behavior change related to NCDs (Table 1).

**Search terms and inclusion and exclusion criteria for the literature search performed to identify the features associated with higher app usability, engagement, and behavior change.**

**Key search terms**

- “Mobile applications” or (“mobile” OR “portable” OR “tablet” OR “smartphone” OR “health”) “App” OR (“smartphone-based”) AND “Motivation” OR “continuous use” OR “success” OR “failure” OR “sustained use” OR “adherence” OR “compliance” OR “engagement” OR “utilization” OR “uptake” OR “motivation” OR “health management” OR “health behavior” OR “lifestyle change”

**Inclusion criteria**

- Apps used on mobile devices (mainly phones and tablets), by healthy people and patients with chronic conditions and having functions that are described in detail. Studies should report on apps that require (at least in part) manual input by the user, monitor and display objective health parameters, provide personalized output, and report outcomes with a causal relation to app functions (ie, there is an explanation on how a certain app function affects the outcome). For randomized controlled trials the study period had to be at least 6 months.

**Exclusion criteria**

- Studies focusing on cost-effectiveness or acute conditions, studies that report only outcomes without any causal relation to app functionalities (ie, there is no explanation on how a certain app functionality affects the outcome), studies without a detailed description of the app used in the trial and economic studies. Randomized controlled trials of <6-months duration. Studies merely investigating drug adherence. Furthermore, studies reporting the following conditions were excluded as they were not in line with the WARIFA concept of chronic conditions: *acute illness, infectious diseases* (eg, *HIV* or *TBC*), *complex psychiatric disorders*, (eg, *psychoses*), and *pregnancy-related gynecological conditions*.

**Table 1:** Health app features associated with higher usability.

Study	Aim of the app	Criteria	Features associated with usability	Comments
Angelini et al [40], 2019	Diabetes management	User perception	Photographs Automatic recording (GPS tracking, heart rate monitor, and pedometer) appears to be more useful than manual recording.	Systematic review Photographs of meals increase user understanding and health care professionals can monitor eating habits. Some apps use image processing to analyze consumed carbs.
Fu et al [41], 2017	Diabetes self-management	Usability scales	Manual data entry restricts usability. Features based on real-time feedback are beneficial for glycemic control. Combination of app with other components improves glycemic control.	Systematic review Most studies analyzed were shorter than 6 months in duration. Many interventions have several additional components (interaction with social forum or health care professionals).
Alfonsi et al [42], 2020	Carbohydrate counting in type 1 diabetes	Qualitative interview Acceptability (E-scale) Satisfaction Effectiveness	Simplicity Clear graphs Clear instructions	Randomized controlled trial The efficacy and acceptability of an app for counting carbohydrates through images was studied in young people.
Kosa et al [43], 2019	Dietary management in chronic kidney disease	Ease of use (qualitative)	Food icons	Systematic review Food icons are helpful in monitoring dietary intake.
Liu et al [44], 2016	Measurement of food contents in the diet	Ease of use (qualitative) Efficiency (response time)	Interactive photo interface	Randomized controlled trial Three free visual aids for the estimation of food intake in college students were compared: Interactive Photo Interface, Sketch Based Interface, and Life-Size Photographs (control).
Liu et al [45], 2020	Measurement of food portion size	Usability scale	No difference between the 3 ways of estimating portion size	Randomized controlled trial Three different ways of measuring portion sizes of food were compared: keyboard-based (weight, volume, or home measurements), photo-based, and gesture-based (gestures or finger movements on the screen to describe volume).
Liu et al [46], 2020	Measurement of food portion size for the elderly	Usability scale Precision Effectiveness Efficiency User perception	Voice-only function	Randomized controlled trial Two forms of food registration were compared in users aged 60-90 years: voice-only vs a combination of voice, buttons, and touchscreen.
Mauch et al [47], 2021	Healthy meal planner	Usability scale Self-reported engagement	Effort-saving features Less manual input Customization Efficiency	Randomized controlled trial Five commercially available healthy meal planners were compared.

#### Challenges to Usability

A key challenge for the usability of mHealth apps is *accessibility.* Even though technology is increasingly present, smartphones and internet connections are not available everywhere. Moreover, the costs associated with their use may also be a hurdle, even when they are technically and geographically accessible. Other challenges are related to language, digital, and health literacy; physical limitations such as blindness; and the lack of awareness of safe and efficient mHealth apps. To address all these challenges, different factors need to be considered when designing these apps [47]: *education*, *payment models*, *functionally diverse users*, and *support structures*, which are especially important given their potential impact on other factors. Furthermore, as the complexity of mHealth apps increases, so do the support needs of patients and clinicians.

#### Assessing Usability of mHealth Apps

Usability is a composite of several attributes related to how easy, productive, and pleasant it is for users to interact with a system. Although there are no standardized approaches to measure the usability of a system, several scales have been developed based on these attributes. In a recent systematic review [48], 4 scales were selected as the strongest available options, based on generalizability, attribute coverage and quality: the *System Usability Scale (SUS)* [49], the *Questionnaire for User Interaction Satisfaction* [50], the *Post-Study System Usability Questionnaire* [51], and the *Computer-System-Usability Questionnaire* [52]. The SUS, which scored the highest, includes the 3 attributes defined by the ISO (effectiveness, efficiency, and satisfaction) and 2 of those defined by Nielsen (learnability and satisfaction). The SUS is particularly useful for the comparison of different technologies intended for the same purpose because it is method-independent; it was designed more than 25 years ago and has been successfully used to evaluate hardware, software, websites, and operating systems [49].

The more recent *mHealth App Usability Questionnaire (MAUQ)* [53] was specifically developed to measure the usability of mobile apps and is strongly correlated with the SUS. Four versions of the MAUQ exist, each of which is designed for a specific type of user (patient or health care provider) and the type of app (standalone or interactive with the health care system). Despite its novelty, the MAUQ has already been incorporated in the assessment of mHealth apps focused on NCDs, such as breast cancer [54], and their risk factors, including alcohol consumption [55] and hypertension [56]. The main challenge facing the assessment of mHealth usability is the *lack of standardization* [48]. Because usability is a complex term encompassing several attributes, it has frequently been misunderstood and only partially assessed. Some assessments are based on validated questionnaires, whereas others use ad hoc questionnaires, semistructured interviews and qualitative research. It is necessary to create better models to understand usability and to develop comprehensive frameworks for the development of mHealth solutions.

### Engagement and Behavior Change Through mHealth Apps

#### Definitions

*Engagement* with a system can be understood as the *time spent by a user interacting with it* and the extent to which a user is willing to continue using it. Engagement has been evaluated based on both single-session length and repeated use over time. There is a growing body of evidence that mHealth apps can be an effective way to promote the self-management of NCDs. However, observational, real-world studies suggest low user retention (0.5%-29% after 6 weeks of exposure to the system) [57]. A recent systematic review and meta-analysis showed an average dropout rate of 49% in real-world, observational studies and 40% in randomized controlled trials [58].

Behavior change is the final aim of most mHealth apps for the prevention and management of NCDs, most of which are directly caused or influenced by lifestyle choices. Healthy eating, physical activity, avoidance of alcohol and tobacco, and protection from dangerous sun exposure are examples of lifestyle choices that can be improved by mHealth apps. However, there are mixed results on the effectiveness of mHealth apps in changing behavior. For example, healthy eating apps focusing on the control or restriction of unhealthy foods were not effective, whereas apps focusing on selecting healthy foods (increasing vegetables and reducing salt consumption) were effective [4]. There is also evidence of a change in the total number of daily steps and a reduction in sedentary time associated with the use of mHealth apps, although no change was found in moderate- or high-intensity physical activity [4].

#### Features Associated With Increased Engagement and Behavior Change

Several *participant-dependent features* have been associated with lower dropout rates [58-60], including younger age, higher health and digital literacy, postgraduate education, poorer self-perceived health, healthy eating at baseline, and being the subject of multiple interventions. The dropout rate is also lower for mHealth apps addressing chronic metabolic diseases than for those addressing nonchronic metabolic diseases [58]. Some mHealth app features have also been associated with increased engagement: *understanding users’ needs and expectations and allowing them to personalize the system* (providing in-app “how-to” guides), *addressing privacy and credibility* (offering contact with health care providers or in-app guidance and support), *minimizing maintenance needs* (cost, energy consumption, and need for manual data entry), and *implementing psychologically based theoretical models and techniques that enhance engagement* (goal setting, tailoring of content toward the goals, feedback in the form of reminders or motivational messages, monitoring progress with graphs and variables, task reminders, recognition of achievements, gamification, and social interaction).

Engagement and behavior changes are clearly related to usability. Ideally, an mHealth app with high usability will lead to increased engagement and, through user interaction with the system, behavior change. Therefore, features promoting usability and engagement should, in theory, promote higher rates of successful behavior change. Several features have been directly associated with behavior changes, including *cognitive behavioral therapy*, *goal setting*, *real-time feedback*, *rewards*, *use of data in social networking*, *easy data collection* (by minimizing manual data entry), *combination with other components of therapy or education outside the system* (usually interactions with health care providers), *social interaction and interactive communication among users and/or with the system*, *reminders*, *gamification,* and *journaling* [12,17,61-66]. As in the previous section, a literature review (Textbox 3) was performed to identify the mHealth app features associated with increased engagement and behavior change (Multimedia Appendix 1).

#### Assessing Engagement and Behavior Change

As with usability, the assessment of engagement has not been standardized. Engagement criteria vary widely from study to study and include different metrics, such as *number of logins*, *frequency of use*, *data entry*, *duration of use* (total or by session), *task completion*, or *self-reported use*. Most metrics count the total number of participants (ie, the denominator in the fraction dropouts/total); however, this is defined differently in different studies and can include all randomized participants in a trial, only those included in the intention-to-treat analysis, only those downloading the app, or only those logging in [58]. Behavior change should be measured in terms of the clinically significant variables that the mHealth app was designed to impact. It is closely related to the clinical usefulness of the app and needs clinical studies to be properly evaluated.

### The Role of AI in Medicine

#### Personalized Medicine Based on AI

In current clinical practice, treatments for many diseases are prescribed based on the patient’s phenotypic features. Personalized medicine aims to prevent diseases and improve their treatment based on the genetic, phenotypic, environmental, and lifestyle characteristics of each individual. The combination of different data sources (*digital medical records*, *omics*, *wearables*, and *sensors*) with AI algorithms (based on both *machine* *learning* and *deep learning*) is leading to promising results [7,8].

Chronic disease management involves routine monitoring and recommendations for patients, where AI can be used as a computer-based medical assistant. This has been investigated in several diseases: in diabetes for monitoring food consumption, glucose concentrations, and physical activity; in cardiac diseases for diagnosing atrial fibrillation through electrocardiogram sensors and improving diagnosis based on cardiovascular imaging; and in lung diseases for predicting chronic obstructive pulmonary disease, pneumonia, and asthma by analyzing sound recordings of the person’s breathing [8]. Nonetheless, AI is not meant to replace the physician but to be used in conjunction with the physician’s own knowledge to improve diagnosis and decision-making [7]. For example, AI can assist cardiologists in making more personalized clinical decisions for their patients, based on the large amount of data generated by echocardiographic systems [67]. In addition, in a study by Han et al [68], the combination of AI and specialist expertise led to a 12% improvement in diagnostic accuracy when distinguishing skin cancer lesions. The algorithm assists the physician with ambiguous cases using all images, while the physician is able to easily identify shaded and blurry images, improve image quality before using the AI algorithm, and minimize possible algorithm errors. These findings suggest that the optimal role of mHealth apps may be as an ancillary tool to assist physicians in routine clinical practice.

The benefit of AI has been demonstrated in several NCDs, including diabetes [69], where it has been used at diagnosis to distinguish between diabetic and nondiabetic individuals, to predict microvascular complications (retinopathy, nephropathy, or neuropathy) [70], and to evaluate the importance of each clinical variable known to be predictive of diabetes, for example, by performing a feature selection analysis, reducing data set dimensionality, and removing features without relevant information [71]. Furthermore, risk prediction models for diabetes have been proposed, based on a combination of clinical knowledge and AI methods [72].

**Figure 2:**
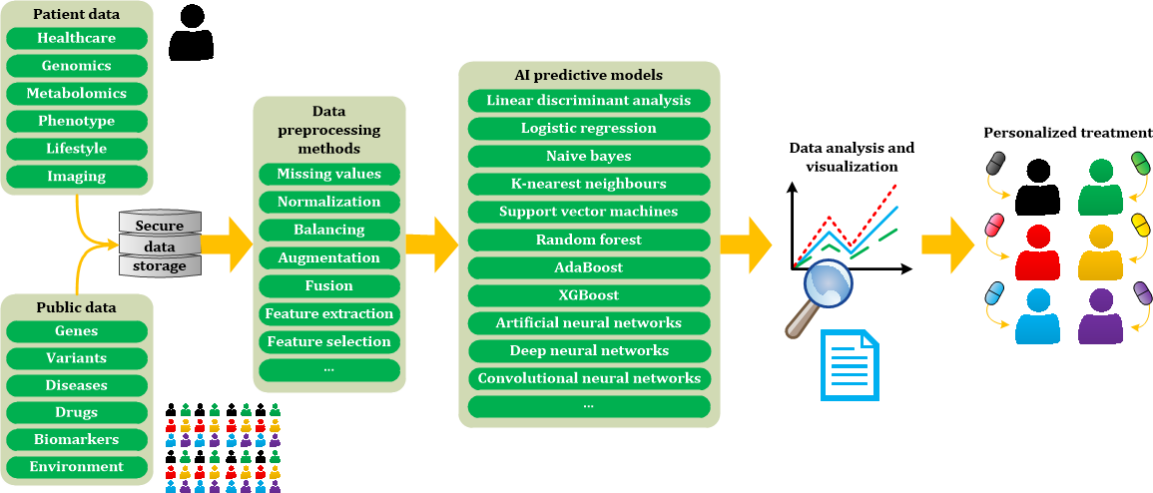
Block diagram of common workflow to apply data analysis and artificial intelligence methods for personalized medicine.

Figure 2 shows a block diagram of the common workflow for using big data and AI algorithms in personalized medicine. Several patient data sources were combined with public data from different large populations. These data are stored in secure servers for later application in different preprocessing methods, such as missing value treatment, data normalization, data balancing and augmentation, data fusion, and feature extraction and selection. These preprocessed data are then used as input for AI predictive models [73], such as linear and nonlinear *machine learning* approaches (linear discriminant analysis, logistic regression, naïve Bayes, K-nearest neighbors, support vector machines, random forest, AdaBoost, and XGBoost) or *deep learning* (artificial neural networks, deep neural networks, and convolutional neural networks). It is important to have large amounts of correctly labeled data to create a gold standard that can be used to train so-called *supervised AI algorithms* [74]. A lack of labeled data can be partially solved in some cases by using *unsupervised algorithms* that do not require the input data to be labeled to differentiate between separate groups or clusters [75]. In addition, the use of *semisupervised algorithms* can generate mathematical classification models based on partially labeled training data [76]. Finally, the output results were visualized and analyzed to achieve personalized treatment and recommendations for the patient.

#### AI-Based mHealth Apps: Current Scenario

AI-based mHealth apps can be used for remote monitoring of individuals affected by chronic diseases, reducing cost and time to diagnosis, and improving health care delivery [9]. In diabetes, for example, AI combined with medical devices, wearables, and smartphones can help physicians and patients, improving risk prediction and diagnostic models, thus enhancing treatment personalization and self-management of the disease [77]. In a 6-week study, an AI-based advanced bolus calculator for type 1 diabetes was shown to be effective as a support system for self-management of this disease, with no hypoglycemic episodes observed in patients using this device [78]. An algorithm that is computationally compatible with low-energy wearables, another key element in mHealth [9], combines metabolic, nutritional, and lifestyle variables to predict not only the onset of type 2 diabetes but also the underlying metabolic and inflammatory processes of the disease [79]. A simple AI-based mHealth app can detect if the user is “out of danger,” “in danger,” or “prediabetic” [80] based only on the user input of 4 variables: BMI, age, gender, and family history of diabetes. In addition to the results, the app recommends preventive actions (eg, *Result: You have a high probability of being in a prediabetic stage*; *Suggestion: Consult your doctor and test HbA1c and sugar levels*). In gestational diabetes, several mHealth apps have been developed to record patient information and provide generic advice and recommendations [81]. However, there are hardly any tools merging mHealth and AI that have been created to empower women with gestational diabetes and aid clinical decision making by both health care professionals and patients. Although there are several mHealth apps based on AI algorithms for diabetes, there is still much to do in this field, combining mobile technologies and AI to enable efficient support for clinical decision–making, self-management, and personalized treatment [81].

In dermatology, the application of AI algorithms to detect skin cancer through image analysis has been addressed typically in two ways: (1) preprocessing the images, applying feature extraction, performing region-of-interest segmentation and data augmentation, and classifying the lesion with *machine learning* or (2) extracting features and classifying the images automatically using *deep learning*.

Many skin cancer apps are currently being used, 235 of which were introduced from 2014 to 2017, and several AI-based mHealth apps deal with skin cancer detection and classification. However, as shown in a study by Takiddin et al [82], the reliability of the performance results (commonly with high accuracy scores) is controversial, because the algorithms are trained using relatively small data sets with limited diagnostic classes. In addition, many different techniques and imaging modalities have been used and different performance metrics have been reported, which hinders a fair comparison of different studies. For example, using *machine learning*, up to 98.92% sensitivity and 99.41% specificity have been reported [83], but extensive image preprocessing is required to achieve competitive results, which might require the use of *deep learning*. Moreover, these results are the best-case scenario, but the performance is expected to decrease with day-to-day use [84]. Using *deep learning* and skin lesion images captured using a smartphone camera in a large study (23,190 participants and 73,255 images), Sangers et al [85] reported 95% sensitivity and 78% specificity [85]. However, this study also revealed low user engagement: although more than 2 million people were invited to participate, only 2.2% (47,879) downloaded the app and created an account and only 1% (23,190) made at least one assessment using the app. One reason could be that even if store-and-forward teledermatology is well-accepted by patients and caregivers [86], trust in the diagnosis or recommendations from an AI-based app is low [87], and medical services provided by a physician might be more familiar to patients. Furthermore, apps such as MelApp and MoleDetective have been withdrawn from the market, as the US FTC fined them for “deceptively claiming that apps accurately analyze melanoma risk,” which may have negatively affected the final user perception of skin cancer apps in general [88].

Another issue with skin cancer apps is the importance of image quality. Obtaining clear images is complicated even under controlled conditions [68], and they are especially difficult to process if these images are shaded or blurry or involve “large lesions, erosive surface of ulcerated tumors, mottled skin, lesions in skin folds, tanned skin, or multiple lesions in close approximation’ [88]. Therefore, patients may require training to take photographs. Nonetheless, digital photographs have been analyzed using AI to distinguish between benign and malignant moles [89]. Although melanoma has been the main focus in this field, as it is likely to metastasize and is known to cause more than 80% of skin cancer deaths in fair-skinned populations [90,91], nonmelanoma skin cancers are also being analyzed using AI [92]. The ubiquity of mobile devices, which in most cases have an integrated digital camera, provides an opportunity to establish teledermatology as a common practice [93,94]. A systematic review [86] summarized its benefits and limitations (Textbox 4).

In addition to their use in the diagnosis of skin cancer, many AI-based mHealth apps can perform other tasks, such as assisting in self-examination, tracking the evolution of suspicious lesions, and store-and-forward teledermatology, which allows the transmission of images and text to support physicians in remote consultations [88].

Other uses of AI-based mHealth apps have also been assessed, such as measuring patient outcomes after surgery [95] or managing public health challenges such as COVID-19 [96]. These examples represent a small portion of the vast dimension of solutions possible with AI-based mHealth apps, which could improve global health, especially for citizens living in resource-limited areas.

**Benefits and limitations of teledermatology.**

**Benefits**

- Quicker identification and management of potential skin cancer lesions
- Reduction of unnecessary visits with a specialist
- Higher patient engagement in follow-up

**Limitations**

- Loss of human contact between patient and physician
- Loss of palpation as part of the examination, with resulting loss of potentially important clinical information
- Absence of a consistent data security policy

### Tackling Challenges in the Design and Implementation of mHealth Apps

#### Overview

Although it is generally accepted that mHealth apps have great potential to improve the health of individuals in a personalized, satisfactory, and efficient manner, the evidence supporting the use of mHealth apps is still weak, and studies have reported varied results regarding engagement and behavior change [58-60]. It is clearly necessary to standardize the measurement of outcomes and find the optimal set of tools to enhance and maintain engagement and behavior change in the long term. In this scenario, AI can help by improving personalization and adapting feedback and rewards, although there are several challenges regarding AI implementation in medicine that must be addressed first.

#### Meeting Users’ Needs

End users’ NEPs influence their perception of, engagement with, and clinical impact of any software or system, making them central to the design and use of mHealth apps. To ensure that users’ NEPs are integrated in an mHealth app, strategies to improve personalization and engagement are needed. Although terms such as *user-centered, human-centered*, and *participatory design* are frequently used, standardized definitions of these terms are lacking, though certain characteristics are generally accepted. *User-centered design* (or *user-driven development*) is a framework for the development of a product or service in which usability, user circumstances, environment, tasks, and workflow receive close attention at each stage of development, usually through testing by potential users. *User-centered design* is based on the understanding of users and their NEPs and has been shown to increase the usefulness, usability, and user satisfaction of a product [97]. ISO 9241-210:2019I defines *human-centered design* as “an approach to interactive systems development that aims to make systems usable and useful by focusing on the users, their needs and requirements, and by applying human factors or ergonomics, and usability knowledge and techniques. This approach enhances effectiveness and efficiency, improves human well-being, user satisfaction, accessibility, and sustainability; and counteracts possible adverse effects of use on human health, safety and performance.” The terms “user-centered design” and “human-centered design” are often used interchangeably, although user-centered design may be a less emotionally empathetic approach, focusing on the tangible ways users interact with a system, whereas human-centered design focuses more on integrating the emotional and psychological preferences of the user. *Participatory design* (also known as *cooperative design* or *co-design*) focuses on involving all stakeholders of a given product, system, or service (employees, partners, customers, citizens, and end users) in the developmental process to ensure that the final product meets their NEPs. It has been applied in many different forms, ranging from the so-called consultative design, which limits user input, to the consensus design, which gives users full participation in the shared responsibility of the final outcome [98]. Finally, it may be useful to distinguish between participatory design and cocreation. Both have in common the involvement of potential users in the development process to ensure that their NEPs are considered, and both should help increase user engagement and adoption of the mHealth solution. However, in participatory design, the design team usually takes the lead by creating a solution that is later presented to potential users. Meanwhile, cocreation strives for more equal collaboration between potential users and the design team, with greater emphasis on user empowerment and a sense of ownership of the solution. Despite these definitions, it should be recognized that the terms are often used interchangeably or with different connotations depending on the setting [99,100].

#### Cocreation and Individual Goal Setting

A *cocreation* framework for the development of mHealth apps can help ensure that users’ NEPs are considered during the design process [58,66,101-103]. *Co-creation* treats end users as experts and incorporates their points of view, even in the early stages of development. For example, when developing massive open web-based courses to improve the digital health literacy skills of European citizens, the IC-HEALTH (Improving digital health literacy in Europe) project [104] adopted this approach and used Communities of Practice (CoPs) to facilitate cocreation. CoPs comprise individuals who share an interest in a particular subject and extend their knowledge of that subject by interchanging ideas and experiences. In the health field, CoPs may include healthy individuals, health-conscious persons, patients, health care professionals, caretakers, other stakeholders, and members of the project team developing the system or app. Shared learning and group aid are central to this concept, which often follow an iterative pattern of user input, development, user review, and continued improvement and development. Textbox 5 shows the steps involved in developing an mHealth app through *cocreation*.

*Cocreation* can also help identify the main obstacles users encounter when using an app. Addressing these barriers during the development of an app will lead to a more user-centered approach. In a recent systematic review [105] of 28 studies published between 2012 and 2019, the potential barriers to the use of dietary advice apps were classified into 4 main categories (Textbox 6).

*Cocreation* also enhances another key aspect of personalization, *goal setting*, which has been extensively studied as a factor influencing engagement and behavior change [67-71]. Evidence suggests that goal setting should be explored by users rather than imposed on them [106-110]. The effectiveness of an mHealth app would improve if users were asked about their health aims so that these aims could guide their behavior change rather than sending messages telling them what should be done. In this way, the overlap between the user-identified “internal goals” and the app-identified “external goals” can be determined, and recommendations for change can remain focused on these overlapping goals. If an external goal is found to be of paramount importance but has not been identified by the user, the app offers information to guide the user in making the best decision. However, it is ultimately the user who will consider their own values and needs and finally decide what to do.

**Steps involved in the cocreation-based development of an mHealth app.**

**Steps involved in cocreation**

- Identification of common objectives shared by all components of the Communities of Practice (depending on the objectives of the app)
- Formulation of the pros and cons of the proposed means of reaching the objectives
- Generation of ideas on how to design the app so it achieves the objectives, while best meeting the needs, expectations, and preferences of its target population
- Continuous evaluation and validation of the resulting software, as well as the adoption of the new concepts generated in previous steps, thus creating a cycle of iterations that closes the gap between the envisioned and real app, which will finally achieve the initial objectives

**Main obstacles to usability of dietary advice apps [105].**

**Obstacles related to the individual user**

- Type of goals and consequences of attaining or abandoning the goals
- Level and evolution of motivation
- Fit between the user’s routines and app use
- Lack of awareness or knowledge of the apps and their capabilities

**Obstacles related to technology**

- App features
- Usability
- Reliability
- Response times
- Technical issues
- Financial costs

**Obstacles related to positive or negative outcome**

- Cognitive changes
- Emotional changes
- Behavior changes
- Health changes

**Obstacles related to the social environment**

- Recommendations for use
- Possible interactions

#### Gamification and Social Networking

Gamification in the context of health behavior exists at the intersection of *games, persuasive technology*, and *personal informatics* [111]. *Games* are meant to be engaging and enjoyable, and their goal in an mHealth app is to drive better health-related outcomes through education, training, and behavior change [111]. *Persuasive technology* is a broad term that includes software for monitoring and managing users’ health and well-being. *Personal informatics* is a tool specifically aimed at collecting and reflecting data on user [111,112]. Gamification in mHealth apps may be defined as the use of game design elements to facilitate user engagement with the app and promote behavior change. Most gamified mHealth apps focus on chronic disease management and rehabilitation. A systematic review [112] has summarized the main advantages and limitations of gamification for mHealth apps (Textbox 7).

Social networking in mHealth is based on the idea that group social interactions affect individual behavior. Social networking focuses on the role of a group as a source of support and motivation for individuals. Some articles within the health field have shown that group interventions can improve health outcomes compared with individual interventions [113-117]. Some challenges arise around the use of social networking such as (1) the need to define what groups benefit from a social element; (2) the need to understand group dynamics such as group size, leadership, attributes of the participants, group relationships, and others to ensure the network creates a positive effect; (3) the possibility of technological challenges for its implementation (is it possible to use existing and popular social networks [Facebook, Instagram, Twitter, etc]? Should a new one be created specifically tailored to the NEPs of the expected end users?); and (4) privacy and security of health information are especially important aspects of any mHealth solution, and social networks pose a new layer of danger to plan for [118].

**Main advantages and limitations of gamification for mobile health apps [112].**

**Advantages**

- Sustains user engagement with the app
- Increases user compliance with health interventions and empowers the user
- Confers positive emotional states and elevates user satisfaction and self-esteem
- Enhances intrinsic and extrinsic motivation
- Makes healthy behaviors understandable and enjoyable while promoting them
- Can help to create social support networks for users

**Limitations**

- Effectiveness not clear when it relies only on one game element
- No unified framework for evaluating principles and outcomes
- Engagement over time may decline despite gamification
- Gamification elements may be perceived as meaningless, unhelpful, or exaggerated
- Gamification solutions not user-centered if they overlook demographics of potential users
- May suffer from reduced performance and credibility if health care professionals are not included in its development

#### AI Implementation in mHealth

AI is emerging as a powerful tool that can transform global health. However, several challenges must be addressed [119].

**Figure 3:**
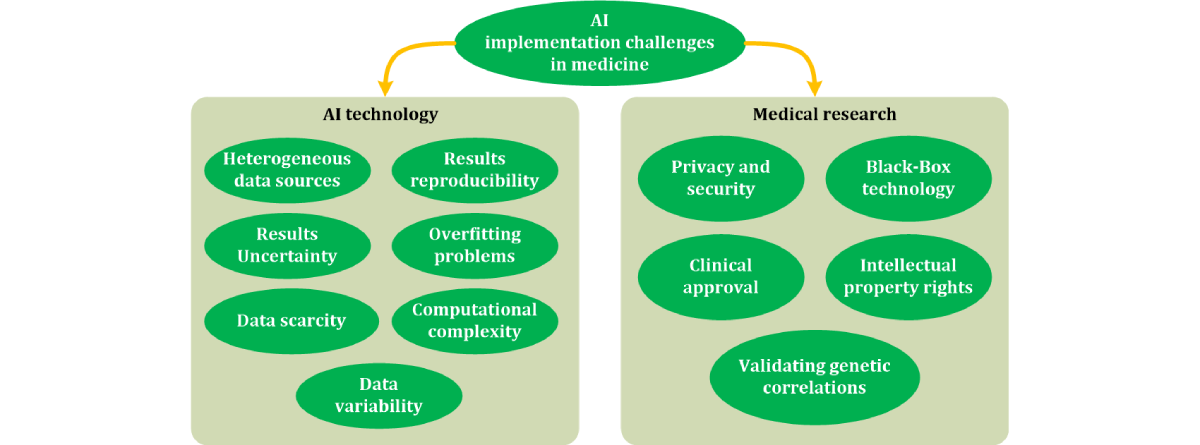
Challenges for artificial intelligence (AI) implementation in medicine [11].

One of the limitations of the use of AI-based mHealth apps is that AI is incapable of critical thinking and thus cannot perform a cognitive and epistemic analysis of the results obtained [10]. Therefore, their validity must be evaluated and critically appraised by specialists, especially to avoid bias. According to a comprehensive review by Zhang et al [11], AI-based personalized medicine faces 2 main challenges (Figure 3): *those related to AI technology itself* and *those stemming from the nature of medical research* (Textbox 8).

As illogical as it may seem in this information age, open and reliable data are scarce, mainly because of the cost of collecting data and the ethical issues associated with managing private clinical data [120]. AI algorithms must be fed with large amounts of data to perform their tasks properly. When using small databases, these algorithms tend to overfit their training data and do not generalize to the entire population [121]. *Synthetic data generation*, also known as *data augmentation* [122], can mitigate the impact of this obstacle [123]. In recent years, research in this field has increased, with a particular focus on medical problems.

Traditional computational models based on ordinary differential equations have been used for synthetic data generation [79]. For example, simulated data have been used together with AI algorithms to assess the risk of type 2 diabetes. A comparison of 5 observational studies analyzed the consistency of the conclusions extracted from simulated data compared with those extracted from real data [124] and concluded that synthetic data provided a close estimate to real data in the shaping of clinical hypotheses. Rankin et al [125] assessed the effectiveness of AI classification by comparing models trained with real data and those trained with synthetic data generated from 19 different data sets using 3 different synthetic data generation techniques. In general, the classification performance was slightly reduced (1%-8%) with the synthetic data compared with the real data. Bayesian networks have demonstrated that they preserve the properties of the original data and AI classification performance for different diseases, including diabetes [126]. Generative adversarial networks are an emerging field in synthetic data generation based on neural networks and have been successfully used. Indeed, a higher accuracy is achieved in diabetes classification when models are trained by combining real and synthetic data than when using only real data [127].

Synthetic data generation can also help organizations work with data that faithfully emulate real patients, accelerating the development of AI tools that can improve clinical practice without compromising patient privacy. Current anonymization approaches may contain enough information to reidentify individuals based on the combination of residual information contained in the anonymized data set and other data sources, such as social media platforms. The use of synthetic data for training AI algorithms could be an efficient alternative for avoiding these problems [123]. Synthetic data generation is an emerging field that needs to be studied further but has shown promising results in the medical field. Using a framework based on synthetic data generation combined with different machine learning classifiers, Rodriguez-Almeida et al [128] tested 8 medical tabular data sets and assessed the feasibility of using synthetic data to preserve data integrity and maintain classification performance using AI algorithms in the medical domain.

Other challenges facing the use of AI in mHealth apps include algorithm processing time, which is critical for a diagnostic tool that requires rapid results. However, the processing time is rarely reported, nor is the equipment used to conduct experiments [84]. In addition, skin cancer apps face data security issues [86,129]. Indeed, there is no standard approach to *data encryption*, with some apps not encrypting data at all. Moreover, it is common practice for specialists to store medical images of patients on their personal phones [86].

In summary, further research is needed on the use of AI in personalized medicine and mHealth apps. Quality criteria-based policies for sensitive data management and protocols to standardize data acquisition are required to improve performance and increase their use among patients and health care professionals.

The 2021 World Health Organization report on “Ethics & Governance of Artificial Intelligence for Health” [130] identifies the ethical challenges and risks related to the use of AI in the health domain (Textbox 9) and presents a set of recommendations to ensure the governance of AI and maximize its successful implementation in medicine.

**Challenges for artificial intelligence (AI) implementation in mobile health from the perspectives of AI technology and medical research [11].**

**AI technology–related issues**

- Structure and origin of data
  - existence of complex and heterogeneous databases: different data sources do not provide standardized data that could be easily combined
  - uncertainty of laboratory results due to random and systematic errors: the acquisition of high-quality data is sometimes operator-dependent
  - need for large amounts of data for training the AI algorithms
- Architecture of the algorithms
  - lack of result reproducibility: different training processes may provide different prediction results in different clinical settings
  - overfitting problems: AI models may be unable to generalize for large and different populations
  - high computational power and time requirements: due to algorithm complexity and data volume
  - intra- and interindividual data variability

**Medical research–related issues**

- Interdisciplinary nature of research
  - data privacy and security: data from many individuals needed to train the algorithm
- Learning process of deep learning
  - need to avoid black-box technology: need to know why a classification process works or not
  - medical expert evaluation: need for clinical approval and validation of the data patterns identified

**World Health Organization core ethical principles for the use of artificial intelligence in medicine [130].**

**Core ethical principles**

- Protect autonomy
- Promote human well-being, human safety, and the public interest
- Ensure transparency, explainability, and intelligibility
- Foster responsibility and accountability
- Ensure inclusiveness and equity
- Promote artificial intelligence that is responsive and sustainable

### The WARIFA European Project

**Figure 4:**
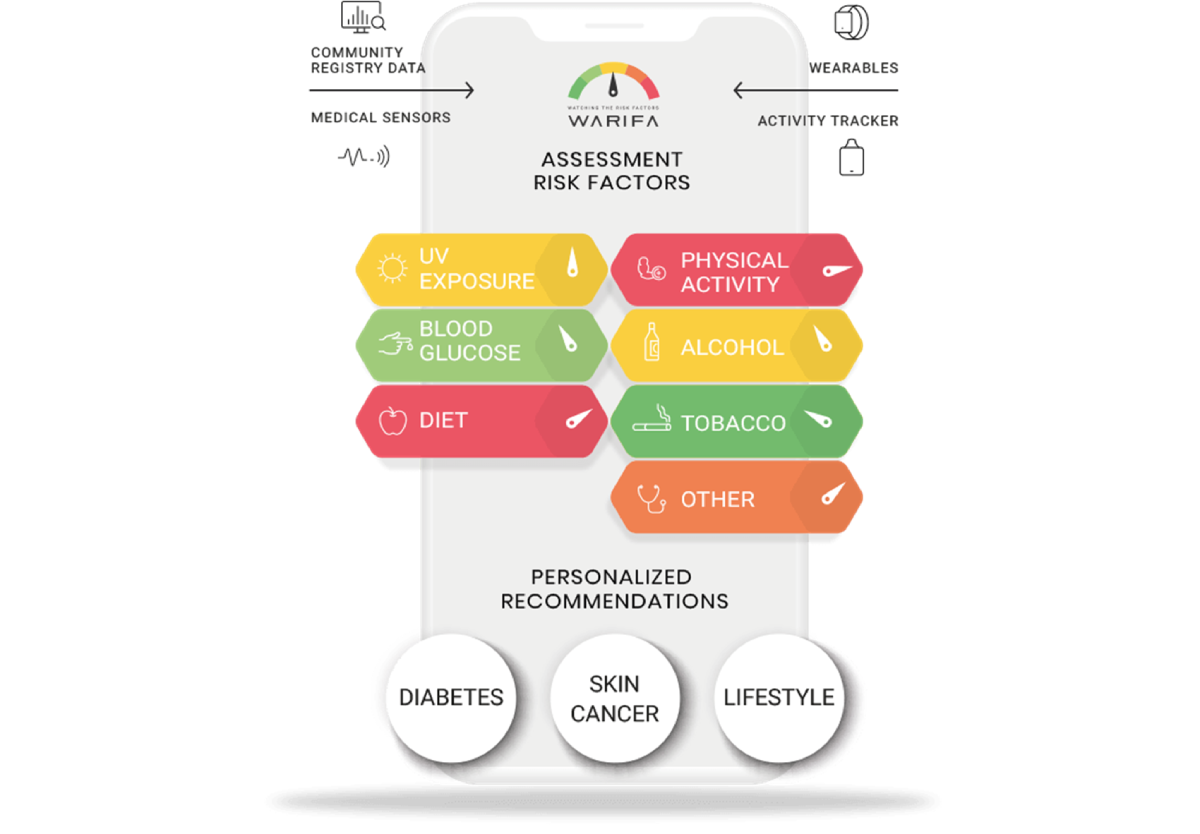
Graphical concept of the Watching the risk factors (WARIFA) App [136].

*WARIFA* is a Horizon 2020-funded EU project (grant agreement ID: 101017385) involving a consortium of 12 partners from 6 European countries [131]. In a proof-of-concept study, WARIFA will develop a technical prototype of a comprehensive AI-based system to provide personalized early risk prediction for multiple NCDs. Individuals will be able to access the system on their smartphones using the WARIFA app. The WARIFA prototype will collect data from several sources, including both user-generated and public data, which will be used to assess the risk of multiple NCDs in the user and offer a personalized set of recommendations on lifestyle, information on health education, and advice on behavior change (Figure 4). For example, to help individuals prevent melanoma or cope with type 1 diabetes, the app will provide personalized recommendations regarding lifestyle risk factors, such as excessive sun exposure, unhealthy diet, and physical inactivity. To maximize the impact of this system, the prototype will be developed within a *cocreation framework* that will include doctors and patient organizations. WARIFA aims to define how health care pathways may be changed to support personalized risk prediction and prevention. The final goal is to empower individuals and increase their ability to take steps to prevent NCDs. WARIFA will thus contribute to health promotion and disease prevention, which will help relieve the burden on health care systems and the economy.

To date, different research steps have been carried out within WARIFA (Textbox 10), which include extensive literature reviews, risk factor mapping, data source and data protocol definitions, front-end development through a cocreation approach, and the development of preliminary AI-based algorithms. In addition, more details can be found in the project deliverables published so far [132] and related scientific publications [133-135].

One of the main barriers the WARIFA project has encountered from the beginning was related to the COVID-19 pandemic, which had a significant impact on project execution. When the project started in January 2021, the pandemic was heavily affecting life in all European countries. Consequently, many planned activities were postponed. During the first 2 years of the project, no physical consortium meetings could be arranged because of European and national travel restrictions. This was alleviated by organizing web-based video gatherings. Some planned interview studies had to be postponed, as national regulations in the participating countries restricted physical contact in the health care sector. In addition, ethical considerations must be considered to avoid unnecessary risks for study participants. Interviews by telephone were considered an option, but it was decided to attempt to get data by surveys instead. We hoped that the interviews would be feasible at a later stage of the project.

As stated before, the development of AI algorithms requires a suitable and large amount of research data. In WARIFA, these data should ideally provide information on the risk factors in question. A perfect single data set complying with these requirements was difficult to find. In addition, privacy and data security aspects require special attention. The WARIFA consortium is expected to overcome these challenges in the last 2 years of the project.

**Research steps carried out in the Watching the risk factors (WARIFA) project so far.**

**Steps so far**

- Mapping the risk factors for the four main noncommunicable diseases considered in the project (diabetes, skin cancer, and cardiovascular and chronic respiratory diseases) in the European countries of the pilot study (Spain, Norway, and Romania).
- Identifying the user and stakeholder needs relevant for the project in the 3 countries under study, supported by patients’ and health care professional associations, and other stakeholder categories.
- Reviewing the literature on app use and usability to summarize the evidence about the usability, use and engagement, and behavior change related to mHealth. This overview has been presented in this manuscript, including the foundations and recommendations set to develop the proposed WARIFA solution.
- Reviewing the literature on health relevant outcomes and the evidence basis on existing validated risk calculators and preventive digital systems for the studied noncommunicable diseases.
- Defining the list of data parameters and data sources to be included in the WARIFA tool in conjunction by clinicians and technicians. This includes manually collected data via questionnaires as well as automatically collected data from wearables, activity trackers, medical sensors, and public databases.
- Analyzing the data security and anonymization measures to be applied in the WARIFA tool, including the definition of the personalization and validation protocol.
- Defining the input and output variables for the WARIFA AI-based tool, as well as the principles and main requirements for creating the user-centered WARIFA app.
- Designing the WARIFA app front-end following a cocreation methodology that involves end users during the entire process of the definition and development. The methodology is based on qualitative research, mainly through the establishment of Communities of Practice and focus group meetings.
- Development of preliminary artificial intelligence–based processing pipelines to combine and process the heterogeneous input data that will be acquired by the WARIFA app.

### Recommendations and Future Perspectives

Here, we summarize the recommendations identified in this work that should be followed for the efficient design and implementation of mHealth apps in routine clinical practice and remote health care (Textbox 11). The mHealth app design should be approached in accordance with the Principles for Digital Development, considering users’ NEPs, providing scalability, sustainability, usability, and accessibility, and addressing data privacy and security. Moreover, existing legal regulations must be followed, and quality can be assessed through quality assurance initiatives, such as the Organization for the Review of Care and Health Apps or Healthy Living Apps. In addition, several tools can be independently used to assess mHealth app quality, such as the Mobile App Rating Scale, App Behavior Change Scale, mHealth service quality, or the DISCERN instrument. Finally, validation before clinical use is paramount, and the recently published V3 validation framework, although proposed for Biometric Monitoring Technologies, is likely to provide useful insights in the field of mHealth app validation.

Despite current guidelines and regulations, we still lack standardized methods to measure the clinical outcomes of mHealth apps and strategies to maintain user engagement and behavior change in the long term. The use of AI can help improve personalization, user engagement, and healthy lifestyle choices. Nonetheless, several challenges must be addressed before AI and mHealth apps can be feasibly integrated into routine clinical practice and remote health care (Textbox 12). The implementation of AI in medicine faces several challenges from both technological and medical perspectives. Technical difficulties related to the protocols for data acquisition and normalization, reproducibility and uncertainty of AI results, and high computational complexity of algorithms require further investigation. Overcoming these obstacles will improve acceptance of this technology in the medical field. Importantly, issues related to user data privacy and security must be addressed to build trust in the use of AI-based mHealth apps. The use of synthetic data to train AI algorithms is a promising solution to partially overcome these issues.

As presented in the previous section, we expect that the WARIFA project will make notable advances in overcoming these challenges in the establishment of AI-based mHealth apps and will provide a framework for future developments based on AI and big data.

**Proposed recommendations for the efficient design and implementation of mobile health (mHealth) apps in routine clinical practice and remote health care.**

**Proposed recommendations**

- Follow the World Health Organization recommendations for the implementation of digital interventions (ensuring stakeholders involvement, health care professional assistance, and network connectivity; providing integration with health systems, data confidentiality, and functional digital devices).
- Guide the design and implementation by the Principles for Digital Development (considering users’ needs, expectations, and preferences through a cocreation framework; providing scalability, sustainability, usability, and accessibility; addressing data privacy and security).
- Meet legal requirements depending on the characteristics of the mHealth app and the region to be implemented.
- Evaluate the quality through different quality assurance tools (Mobile App Rating Scale, App Behavior Change Scale, Healthy Living Apps, Organization for the Review of Care and Health Apps, the Health on Net code, or the DISCERN instrument).
- Validate the quality assurance through the V3 validation framework applied to mHealth apps.
- Ensure usability and accessibility by considering different factors related to the users, such as education, payment models, functionally diverse users, and support structures, while designing the app.
- Assess usability through different measuring scales, mHealth App Usability Questionnaire being the most recent.
- Ensure user engagement and behavior change by developing personalized apps using AI-based algorithms and considering goal setting and gamification.
- Follow the World Health Organization core ethical principles for the use of artificial intelligence in medicine.

**Proposed measures for solving the current challenges of implementing mobile health (mHealth) apps and artificial intelligence (AI)–based solutions in health care systems.**

**Proposed measures**

- Define and implement standardized international certification programs for mHealth apps.
- Design and develop new models for understanding usability and standardized global frameworks for assessing usability.
- Design and develop standardized methods to assess users’ engagement and the clinical outcomes associated to the behavior change induced by mHealth apps.
- Define standardized protocols for data acquisition and normalization to combine different data sources, aiming to reduce uncertainty of results and increase databases to train generalized artificial AI-based models.
- Define standardized protocols and quality criteria-based policies to ensure users’ data privacy and security in AI-based mHealth apps and to assess reproducibility of the results.

## Appendix

Multimedia Appendix 1 Mobile health app features associated with increased engagement and behavior change.

## Abbreviations

AI: artificial intelligence
BioMeT: Biometric Monitoring Technology
CoP: Community of Practice
EU: European Union
FTC: Federal Trade Commission
ISO: International Organization for Standardization
MAUQ: mHealth App Usability Questionnaire
mHealth: mobile health
NCD: noncommunicable disease
NEP: need, expectation, and preference
SUS: System Usability Scale
WARIFA: Watching the risk factors
