# Supplementary material for: Quality, Usability, and Effectiveness of mHealth Apps and the Role of Artificial Intelligence: Current Scenario and Challenges
Source: J Med Internet Res. 2023 May 4;25:e44030. doi: 10.2196/44030 (PMC10196903; doi:10.2196/44030)
Supplement: Multimedia Appendix 1 [file jmir_v25i1e44030_app1.docx]

**Table S1.** Mobile health app features associated with increased engagement and behavior change.

| Reference | Aim of the app | Criteria measuring engagement or behavior change | Features associated with improved outcomes | Comments |
| --- | --- | --- | --- | --- |
| Agarwal et al [68], 2019 | BlueStar: type 2 diabetes self-management: users report glucose readings, exercise, food intake, and app delivers customized, evidence-based messages. | Engagement: entries in app | - Blood glucose tracking feature - Usability evaluated by an adapted MARS^a^. - A qualitative analysis found that the health care provider was an important source of support. | - Multicenter pragmatic randomized controlled trial. - High variability in use by site; higher use might be due to better implementation. - Study participants could not use their own phone. |
| Coorey et al [69], 2018 | Self-management of cardiovascular disease | Engagement: app use frequency, duration, data registration, responsiveness to daily tasks. Self-reported preferences. | - Healthy eating and exercise goal setting - Recognition of achievements - Memory and psychological tasks - Editable self-entered numeric data, reminders and appointments - Motivational messages with opt-out option, user-created and system-generated content responsive to user input - Game-based design techniques - Ability to enter textual and numeric data - Graphic data displays viewable on a smartphone - Cardiac rehabilitation-related app content tailored to stage of recovery - Team-based competition options - No requirement of daily data entry - In-app “how to” guides | - Systematic review - Often low participant numbers, dropouts, and short exposure duration |
| Gong et al [70], 2020; Baptista et al [71], 2020 | Self-management of type 2 diabetes: “My Diabetes Coach,” app with an embodied conversational agent (Laura) providing gamification and human-like features | Engagement: user-reported QoL^b^ and HbA1c^c^ | - Dose-response relationship between the number of chats and change in QoL - >80% of participants considered Laura helpful, friendly, and competent and 72% found her trustworthy. - User self-management style affected app experience. - Interactive voice recognition did not offer any obvious advantages. - Personalization and tailoring were beneficial. | - Randomized controlled trial - Qualitative study reporting user experience |
| Meyerowitz-Katz et al [59], 2020 | Chronic disease self-management | Engagement: drop-out rate | - Varying and tailored messaging - Self-management skills - Contact with health care provider - Guidance/support | - Systematic review - Wide range of diseases - Heterogeneity |
| Monteiro-Guerra et al [72], 2020 | Physical activity coaching | Engagement: not specified | - Authors highlight 3 key aspects: tailoring, based on behavior change theories, gamification | - Scoping review - Theoretical review - No quantification of association/causality |
| Szinay et al [61], 2020 | Promotion of healthy behavior regarding smoking, alcohol, physical activity, and diet | Engagement: number of logins, frequency of use, other measures tracking user engagement | - Psychological capability: user guidance, health information, statistical information on progress - Memory, attention, and decision processes: well-designed reminders, less cognitive load, including automatization of data collection, coping games - Behavioral regulation: self-monitoring, established routines, safety netting - Physical opportunity: availability, accessibility, low cost, interactive positive tone in messages, personalization - Social opportunity: health professional support, community networking, competition - Automatic motivation: feedback, rewards - Reflective motivation: goal setting | - Systematic review - Authors provide a list of recommendations based on their findings to guide health app and portal developers and policy makers. |
| Wang and Qi [60], 2021 | Health issues in general (not specified); Healthy lifestyle promotion; Chronic disease prevention | Engagement: mobile app acceptance and use, user feedback | - Social dimension: social networking increases interest and promotes users’ staying power - Source credibility: published by credible mass media, recommendation by health care professionals or institutions. - Legal supervision. - App design: accuracy, timeliness, and relevance; reminders, notifications, encouragement, follow-up, and goal setting; personalization; gamification; clean and simple interface; efficiency; perceived usefulness; security and privacy; cost - Personalization: self-assessment, feedback, and customization. - Reinforcement: rewards and reminders. - Communication: with peers and health care professionals - Navigation: ease of use, automation, guidance - Credibility: no advertisements, evidence-based, privacy, data protection. - Message presentation: simple, specific, positive, nonjudgmental, gamification - Interface esthetics: attention-grabbing, simple, consistent | - Systematic review - Most articles used qualitative and mixed methods |
| Wu et al [74], 2021 | Management of depression and anxiety | Engagement: duration of use and completion of interventions | - Unexpected negative association between persuasive system design features (primary task support, self-monitoring and personalization) and engagement, as measured by completion rate | - Systematic review - Heterogeneity - No association between engagement and effect on symptoms |
| Fitzgerald and McClelland [62], 2017 | Health issues in general (not specified) | Behavior change: not specified | - Key features; education, personalization, and networking - Most effective: cognitive behavior therapy, goal setting, real-time feedback; collection of data, personalization, use of data in social networking to reinforce desired behavior - Less effective: little involvement by professionals, inflexibility, irrelevant content, poor customization | - Systematic review - Discussion of behavior change theories |
| Fu et al [41], 2017 | Diabetes self-management | Behavior change: HbA1c | - Real-time feedback beneficial for glycemic control - Combination of app with other components improved glycemic control. | - Systematic review - Most studies were <6-months long. - Many interventions had several additional components. |
| Hosseinpour and Terlutter [63], 2019 | Increase in physical activity | Behavior change: physical activity | - Feedback positively affected users’ physical activity. Some studies found negative effect of negative feedback. - Goal setting positive if realistic - Rewards: badges, trophies, ribbons, stars, electronic postcards, encouraging messages, points - Social sharing: sharing with strangers in segregated groups appeared to have mixed effects. | - Systematic review guided by theoretical framework |
| Iribarren et al [17], 2021 | Increase healthy lifestyle and manage disease.  Any disease could be included, but most studies investigated chronic or lifestyle-related diseases | Behavior change: variety of health outcomes | - Interactive communication - Reminders - Gamification - Journaling | - Systematic review - Logistic regression showed nonsignificant positive effect on health outcomes for these features - No data on app use |
| Li et al [64], 2020 | Self-management of hypertension | Behavior change: medication adherence; outcomes related to blood pressure control | - Medication reminders - Interaction with health care professionals - Multiple app functions | - Systematic review - Trials with a tailored frequency of reminders, a patient-physician interactive loop, and multifaceted functions showed a larger overall effect on blood pressure, compared with trials with a fixed frequency of reminders, a noninteractive loop, and a single function. - Not all studies used apps. |
| Peiris et al [65], 2019 | Smoking cessation | Behavior change: number of quitters | - Participants suggested: improve functionality; greater customization of messages; integration with existing social platforms; gamification | - Pilot randomized controlled trial |
| Stuckey et al [66], 2017 | Increase in physical activity | Behavior change: step count, energy expenditure, self-reported change in physical activity | - No specific behavior theory or change technique better than others - App features evaluated: feedback, motivational cuing, information and education, reminders, social support, gamification, goal setting - Behavior change theories evaluated: Five A’s model, learning theory or operant conditioning, self-determination theory, social cognitive theory, social influence theory, theory of reasoned action, transtheoretical model | - Systematic review - Some studies included interactions with health care professionals |
| Tong et al [67], 2021 | Improvement in lifestyle | Behavior change: physical activity, diet, smoking, alcohol consumption | - Automatic data acquisition associated with higher effectiveness than manual entry (but similar in systems that used both). - Interventions mostly personalized their content and rarely personalized other features, such as intervention timing, dosage, or delivery. - Personalization algorithm lacked detail. | - Systematic review - Other modalities included in some studies (websites, e-mails, or interaction with health care professionals) |
| Villinger et al [12], 2019 | Improvement in diet (patients and healthy citizens) | Behavior change: BMI, lipids, nutrition (Healthy Eating Index, total caloric intake, meal types, specific foods, and nutrients) | - Positive effects on obesity, blood pressure, lipids, and nutrition behaviors - No notable effect of specific behavior change techniques - The inclusion of additional treatment components besides the app or the number or type of behavior change techniques did not change effectiveness. | - Systematic review - Overall pooled effect size was positive and significant. - Effects were small but reliable. - Long-term (>6 months) effects were generally small and nonsignificant. |

^a^MARS: Mobile App Rating Scale.

^b^QoL: quality of life.

^c^HbA1c: glycated hemoglobin.
